# Publication of Medical Books in Connexion with This Journal

**Published:** 1841-11

**Authors:** 


### THE WESTERN JOURNAL

Vol. IV.—No. V.

LOUISVILLE, NOVEMBER 1, 1841.

### PUBLICATION OF MEDICAL BOOKS IN CONNEXION WITH THIS JOURNAL.

We ask the particular attention of our readers to the advertisement which our publishers have enclosed in this number. It sets forth, that, under the direction of the Editors, they propose hereafter to publish, in connexion with each number of the Journal, from 80 to 100 pages of some medical work, so as to bring out, annually, two volumes of about 500 pages each. This addition however will be sent to those alone who desire it, and signify that desire in the manner pointed out in the circular; all other subscribers will receive the Journal *only*. Three classes of medical books will be thus published. 1st. Original, chiefly by the professors of the Medical Institute. 2nd. Translations from the French and German. 3rd. English, Scotch and Irish works, not previously reprinted in this country. The two latter classes will generally be accompanied with notes and additions, by some one of the professors. This enterprise will commence with our 5th volume, on the 1st of January, 1842.

The postage on the works thus published, will be for each volume of 500 pages, about 37½ cents to those who live within 100 miles of Louisville, and 62½ to those who reside at a greater distance; but the difficulty which physicians, living in remote places, necessarily encounter, in procuring books from the eastern cities, must, we presume, render this method of obtaining them acceptable. The subscription to the Journal thus enlarged, will be ten dollars a year; for which sum the subscribers will receive four volumes, or about 2000 pages of matter, original or recently published in Europe; an amount sufficient, independent of other purchases, to keep him informed on the progress of discovery and improvement in the profession. The volumes of the first year, will embrace an elementary treatise on Pathology by Dr. Drake.

Such is the plan of our publishers; but they will not enter upon it, unless a sufficient number of subscribers should be obtained; and of course they will not make arrangements for its execution, before they hear from the patrons of the Journal. As it is proposed to commence on the first of January next, we respectfully request our readers to give prompt attention to the circular, that the number of the Journal for that month may not be delayed.

